# GPCR molecular dynamics forecasting using recurrent neural networks

**DOI:** 10.1038/s41598-023-48346-4

**Published:** 2023-11-28

**Authors:** Juan Manuel López-Correa, Caroline König, Alfredo Vellido

**Affiliations:** 1 Universitat Politècnica de Catalunya, Barcelona, Spain; 2 IDEAI-UPC - Research Center, Universitat Politècnica de Catalunya, Barcelona, Spain

**Keywords:** Biotechnology, Computational biology and bioinformatics, Molecular medicine

## Abstract

G protein-coupled receptors (GPCRs) are a large superfamily of cell membrane proteins that play an important physiological role as transmitters of extracellular signals. Signal transmission through the cell membrane depends on conformational changes in the transmembrane region of the receptor, which makes the investigation of the dynamics in these regions particularly relevant. Molecular dynamics (MD) simulations provide a wealth of data about the structure, dynamics, and physiological function of biological macromolecules by modelling the interactions between their atomic constituents. In this study, a Recurrent and Convolutional Neural Network (RNN) model, namely Long Short-Term Memory (LSTM), is used to predict the dynamics of two GPCR states and three specific simulations of each one, through their activation path and focussing on specific receptor regions. Active and inactive states of the GPCRs are analysed in six scenarios involving APO, Full Agonist (BI 167107) and Partial Inverse Agonist (carazolol) of the receptor. Four Machine Learning models with increasing complexity in terms of neural network architecture are evaluated, and their results discussed. The best method achieves an overall RMSD lower than 0.139 Å and the transmembrane helices are the regions showing the minimum prediction errors and minimum relative movements of the protein.

## Introduction

G protein-coupled receptors (GPCRs) are a large and diverse superfamily of eukaryotic cell membrane proteins. They are receptors for a large diversity of extracellular signals including light, pressure, chemical ligands, neurotransmitters and metabolites, among others^1–4^, and play an important physiological role as transmitters of extracellular signals to the cell^5^.

Due to their participation in a wide range of activation pathways and important biological processes and because of their high affinity binding to drugs, GPCRs have become a prime research concern in pharmacology and a major target for drug discovery^6^. In fact, approximately 34% of all the drugs approved by the US Food and Drug Administration^5^ target GPCRs with the aim of either activating (agonist) or deactivating (antagonist) the receptor^7^.

The functionality of proteins is determined by their 3D structural configuration, which varies according to the binding processes of orthosteric and allosteric ligands, the lipidic environment and post-translational modifications^8^. These sources of variability elicit dynamical changes in the GPCR that result in the generation of specific signals.

The understanding of these signal transmission mechanisms in the receptor would provide us with a key to drug development and testing. The GPCR structure includes seven trans-membrane (TM) helices, linked by intra-cellular loops (ICL) and extracellular loops (ECL). All these regions play a role in the activation process^9^, but the TM regions are of particular importance^10^, as they have to undergo a conformational change to transmit the signal trough the cell membrane.

Molecular dynamics (MD) simulations provide a wealth of data about the structure, dynamics, and physiological function of biological macromolecules by modelling the interactions between their atomic constituents. The computer-assisted analysis of MD simulation data should allow the study of the receptors dynamic behavior, particularly in their interaction with drugs. Machine Learning (ML) tools can be particularly efficient in such endeavours.

This study investigates the ability of a recurrent neural network (RNN) model, namely Long Short-Term Memory (LSTM)^11^, to predict the dynamics of two GPCR states and three specific simulations of each one, through their activation path. Most importantly, the relative relevance of different regions of the receptor (TM, ECL, and ICL) for this prediction is also estimated as part of the analysis. A unidirectional LSTM (ULSTM) and a bidirectional LSTM (BLSTM) are used to predict the path trajectories for three types of simulations in the active and inactive states of the ${\upbeta }$-2 adrenergic receptor (${\upbeta }$ 2AR) GPCR. More specifically, these simulations are analysed for the *2RH1* (inactive state) and *3P0G* (active state) structures, both with APO, Full Agonist (BI-167107) and Partial Inverse Agonist (carazolol). In addition, the LSTM variants are compared to other architectures, such as Random Forest (RF) and chains of Artificial Neural Networks (ANN), namely Convolutional Neural Networks (CNN) with LSTM (CNN-LSTM).

Advances in biotechnology, X-ray crystallography, and cryoelectron microscopy (cryo-EM) in recent years have generated an exponential increase in available GPCR simulation data, easing GPCR analysis, visualisation, and data-driven experimental designs^5^. Machine Learning can be used as tool to extract knowledge from complex data and different ML models have successfully been applied to many areas in proteomics, including the MD domain. They have been applied, for instance, to the study of protein pocket dynamics^12^, to enhance sampling^13,14^, and to generate new digital structures^15,16^. Some studies have used ML methods to identify different biological function states from MD conformations to explain the allosteric mechanism. For example, Fleetwood *et al.* (2020)^17^ used ML and statistical approaches (Principal Component Analysis, Random Forest, Autoencoder, Restricted Boltzmann Machine, and Multilayer Perceptron) to analyse conformational changes within the soluble proteins and ligand binding to a GPCR. Zhou *et al.* (2018)^18^, in turn, used Decision Trees and ANNs to classify ligand unbound and bound states from MD trajectories of PDZ2 protein. Both models achieved, in turn, 75% and 80% of predictive accuracy.

Most recent ML-based approaches concern the use of different variants of Deep Learning (DL) methods. Jumper *et al.* (2022)^19^ and Baek *et al.* (2021)^20^ developed, respectively, the very successful AlphaFold and RoseTTAFold models for protein structure forecasting from sequence as input. Other authors have applied CNN methods to the prediction of interactions between proteins^21^, protein with ligand^22,22^, protein folding, protein phosphorylation^23^, and protein structure classification^24^. Notice though that the input data for the CNN are images described as spacial arrays, whereas the characteristics that describe GPCRs conformation are not structured. Hayatshahi *et al.* (2019)^25^ distinguished otherwise similar allosteric states of proteins adopting conventional ML and DL approaches on extensive MD simulations. Plante *et al.* 2019^26^ combined a densely connected ANN and the pixel representation to identify ligand bound and unbound states.

Recurrent Neural Networks are ANNs where connections between points-nodes can create a cycle. This enables them to show temporal dynamic behavior. They have been particularly successful in applications to human language modelling^27^. The LSTM models address a limitation of the RNN architecture, namely its inability to learn information from the distant past, allowing the network to dynamically *learn to forget* old aspects of information. They have been used to mimic trajectories produced by simulations^28,29^, achieving accurate short-term predictions. They have also shown great potential for sequence processing^30^, resulting in a large body of literature studying the trajectories from simulation systems^31^.

In Tsai *et al.* (2020)^29^, LSTMs were used to predict the temporal evolution of chemical/biophysical trajectories. Mohamma *et al.* (2019)^32^ applied these models to find temporal correlations between atoms. Kadupitiya *et al.* (2020)^33^ used LSTM for the numerical integrator that solves Newton’s equations in MD simulations^33^. Other authors have applied LSTM over the low-dimensional molecular simulations to detect rare events in the sequential data^34^. Liang *et al.*^35^ applied them to molecular step-positions forecasting for S-protein on the SARSCoV2 dynamics. Ludwig *et al.*(2022) evaluated the performance of BLSTMs in the task of increasing the 3D spacial resolution of MD trajectories as a data post-processing step.

We carried out a preliminary study using LSTM^36^, in which the best representation of amino-acids in 3D space to predict MD trajectories of a GPCR receptor as a whole was obtained, but, to the best of our knowledge, there is no reported work on MD forecasting by GPCR regions, which is the main concern of the current study.

## Materials

### GPCR MD simulations

The MD simulations used in this study were created in Google Exacycle cloud computing platform^37^ as a way to improve understanding of the drug efficacy at GPCR receptors. These simulations could be incorporated into a valid and functional structure-based drug discovery approach through pathway analysis. The simulations under study were created by Kohlhoff *et al.*^38^ by computing intensively short MD trajectories in parallel on the cloud platform, and are publicly available as source data at the SimTK (https://simtk.org/projects/natchemgpcrdata/) repository. The authors of these short simulations further analysed them by assembling larger trajectories using extensive sampling with Markov state modeling. We summarily describe these larger simulations next according to the description by their authors.

The crystal structure of the membrane for PDB id:2RH1 (inactive) and id:3P0G (active) was created from the OPM database^39^. Inactive (2RH1) and active structures (3P0G) without ligand (APO) in addition to binding of the receptor to the partial inverse agonist and the full agonist (2RH1 with BI-167107, and 3P0G with carazolol)^40,41^.

The structures were embedded in a bilayer of POPC lipid molecules in a orthorhombic box of size 10.0 $\times$ 10.0 $\times$ 8.5 nm. The system was solvated in TIP3P water molecules interspersed with Na+ and Cl- ions for molecular stabilisation with cholesterol and a final ion concentration of 0.15 M.

Protein, water, and ions were parameterized with the AMBER03 force field^42^ and lipids with the Berger unified atom force field. Carazolol and BI-167107 ligands were extracted from the PDB entries 2RH1 and 3P0G, respectively, and parameterized for the general Amber force field (GAFF)^43^ with acpype^44^ and antechamber^45^. For simulations in which the agonist and the partial inverse agonist were switched, the ligand positions were changed after superimposing the two crystal structures using all protein residues with atoms within 6Å of either ligand. The sizes of the resulting molecular dynamics systems range from 58,406 to 59,044 atoms.

The receptor structures of both N- and C-termini were not fully resolved during crystallography. In 2RH1, the structures involve residues from 30 to 342, and for 3P0G, residues from 23 to 344. In intracellular loop 3 (ICL3), between helices 5 and 6, the missing residues are substituted in 2RH1 and 3P0G with T4 lysosomes and a nanobody, accordingly. These residues are 231–262 in 2RH1 and 228–264 in 3P0G. ${\upbeta }$2AR remains functional even in the absence of ICL3.

Hydrogen bonds and hydrogen bond networks enable intramolecular water to act as a facilitator of biomolecule dynamics. During the equilibrium and production experiments, water molecules were able to move freely within the simulation system and enter and exit the receptor during the simulations.

Considering ionic lock formation, a salt bridge between intracellular residues E268 and R131 is a feature of the receptor’s inactive state and disruption of this ionic lock is involved in receptor activation^46^. It has been demonstrated that the inactive state shows a mixture of ionic locks formed and broken at equilibrium^47^.

In the extracellular region, the helical movements extend around the mean helical position. The crystal structures of the active and inactive conformations on the extracellular side are almost identical in the movements of helices 2 and 3 (with a difference of less than 1% or less), while the other five helices are shifted from 0.379 Å (helix 4) to 0.773 Å (helix 1) in relation to each other. The active structure has a more compact helical formation than the inactive structure. During the simulation, helices 6 and 7 were compressed in all systems, while helices 4 and 5 moved slightly outward. Helix 1 showed the greatest relative movement within the simulations, particularly in the inactive structure.

The central region of the transmembrane helix shows the greatest stability compared to the intra- and extracellular sides. This region is usually quite condensed. The most significant distinctions between the active and inactive structures are observed in helices 1, 6 and 7. During the simulation, helix 6 appears to be converging towards the inactive state when the simulation is initiated from the active conformation. Helix 1, on the other hand, moves out in all systems.

The most notable structural differences are seen in the movement of the transmembrane helices in the intracellular region. Helices 6 and 7 are particularly distinct between the active and inactive structures, with a displacement of 6.951 Å and 3.47 Å respectively. Helices 1, 2, and 4 are further away from the center in the active state, while helix 3 is much closer (with a range of offsets from 1.4 to 2.277 Å).

Residues by helix, and limits of the helices by residue id are defined as follows (with residue numbers in brackets): Helix 1 (29–60), Helix 2 (67–96), Helix 3 (103–136), Helix 4 (147–171), Helix 5 (197–229), Helix 6 (267–298), Helix 7 (305–328)^48^.

Although Kohlhoff *et al.*^38^ assembled a larger simulation for the different crystal structures from the short simulations using an extensive sampling with Markov state modelling, the simulations in this study are based on short trajectories which are released at the source repository by the authors. More precisely, the simulations in the study comprise 2, 000 trajectories for each, classified into six types of crystal structure of ${\upbeta }$2AR: APO for the simulations of 2RH1-icl3 and 3P0G-a, Full Agonist (FA) for the simulations of 2RH1-b and 3P0G-b, and Partial Inverse Agonist (PIA) for 2RH1-c and 3P0G-c. The receptor consists of 282 amino acids for the inactive and 344 for the active states. Each trajectory describes the 3D position of the receptor along 28 consecutive time-steps (trajectory length), which are hereon referred to as *frames*. The time elapsed between each frame is 500 picoseconds. Activation and deactivation proceed through multiple pathways and typically visit metastable intermediate states. The simulation data under study, as in Gutiérrez-Mondragón *et al.*^49^, primarily comprise intermediate states of each receptor.

### Structural sequence domains

GPCRs have three main structural regions, namely a seven-helix TM domain, an extracellular domain built by the N-terminus and three ECLs, and the intracellular domain, including the C-terminus and two ICLs^50^.

Table 1 provides a detailed description of each region of the ${\upbeta }$2AR-GPCR receptor under study. The 2RH1 and 3P0G structures contain residues 30-344. Both have gaps in the sequence, where ICL 3 between TM2 and TM3 is replaced in 2RH1 and 3P0G with T4-lysozyme and a nanobody, respectively. These residues are 231-262 for 2RH1, and 228-264 for 3P0G. ${\upbeta }$2AR remains functional even in the absence these regions.

**Figure 1:**
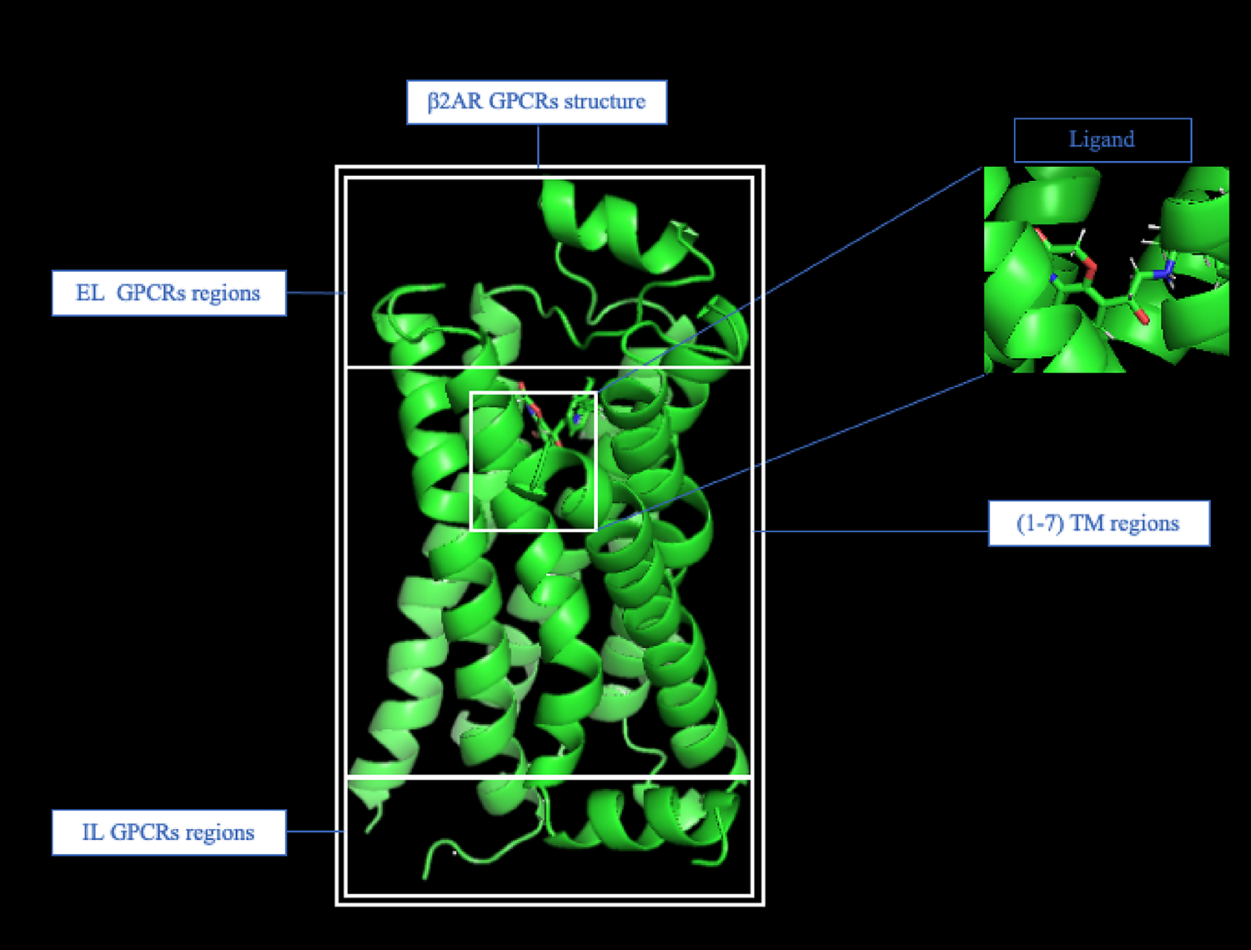
Schematic representation of the ${\upbeta }$2 adrenergic GPCR, including TM, ECL and ICL regions in Full Agonist (BI-167107) trajectory.

Figure 1 represents the common structure of a ${\upbeta }$2 adrenergic GPCR. In it, the 7 TM, 2 ICL and 3 ECL regions are shown. In addition, BI-167107 ligand binding with the protein is displayed in an image inset.

**Table 1:** ${\upbeta }$2AR-GPCRs amino acid distribution by regions for inactive (2rh1) and active (3p0g) states.

Region	Amino acid id
N-terminus	[0–30)
TM 1	[30–61)
IL 1	[61–68)
TM 2	[68–97)
EL 1	[97–104)
TM 3	[104–137)
IL 2	[137–148)
TM 4	[148–172)
EL 2	[172–198)
TM 5	[198–230)
TM 6	[268–299)
EL 3	[299–306)
TM 7	[306–329)
C-terminus	[329–344)

## Methods

### The long short-term memory model

**Figure 2:**
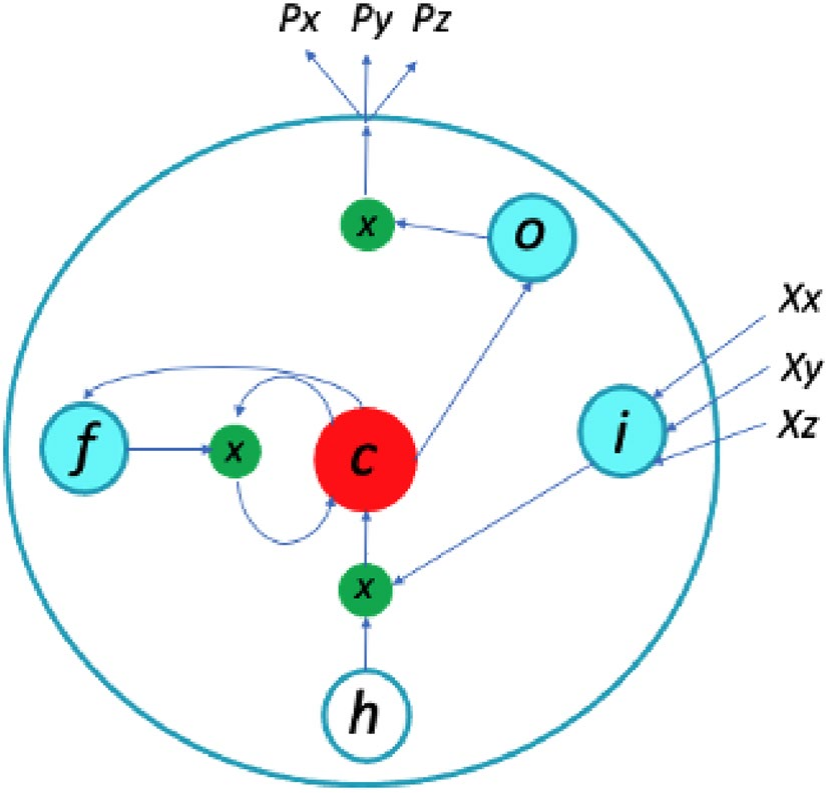
Illustration of an LSTM unit. *X* represent the input data for 3D positions of the amino-acids, and *P* their 3D predicted positions.

LSTM^11^ is a neural network of the RNN family, designed for the analysis of temporal data. A schematic explanation of how LSTM works is shown in Fig. 2. Summarily, LSTM has an input gate *(i)*, a forgetting gate *(f)*, a memory gate *(c)* and an output gate *(o)*. The input gate decides whether to let the incoming signal go through to the memory gate, or block it. The output gate could allow a new signal output, or avoid it trough the memory gate. The forgetting cell is responsible for remembering or forgetting the previous state of the memory gate. The update of memory gate states is carried out by feeding the previous output gate back onto itself by recurrent connections of two consecutive time steps. The reading-and-writing memory cell is controlled by a group of sigmoid gates (*x*). At a given time, the LSTM receives inputs from different sources: the current amino-acid positions $\textit{X}_\textit{xyz}$ as the input, the previous hidden state of all LSTM units (*h*), as well as the previous memory gate state $\textit{c}_{(t -1)}$. Then, the output gate returns the estimated probability of the next 3D amino-acid positions for the sequences (Px, Py, Pz).

In short, LSTM solves one of the limitations of the RNN architecture, namely the inability to learn information from the far past. Therefore, LSTMs are able to accumulate information for a long period of time by allowing the network to dynamically learn and forget old aspects of information. In this work, an LSTM model was trained with 3D position sequences to predict their next movement.

In this paper, ULSTM, BLSTM, as well as CNN-LSTM as a chain of ANNs are investigated. ULSTM works by processing data in the forward direction, while BLSTM processes sequence data in both forward and backward directions with two separate hidden layers^51^. In addition, the LSTM variants are evaluated with other ML approaches, namely RF and CNN, in order to compare the results obtain with model architectures of different complexities.

### Convolutional neural network

**Figure 3:**
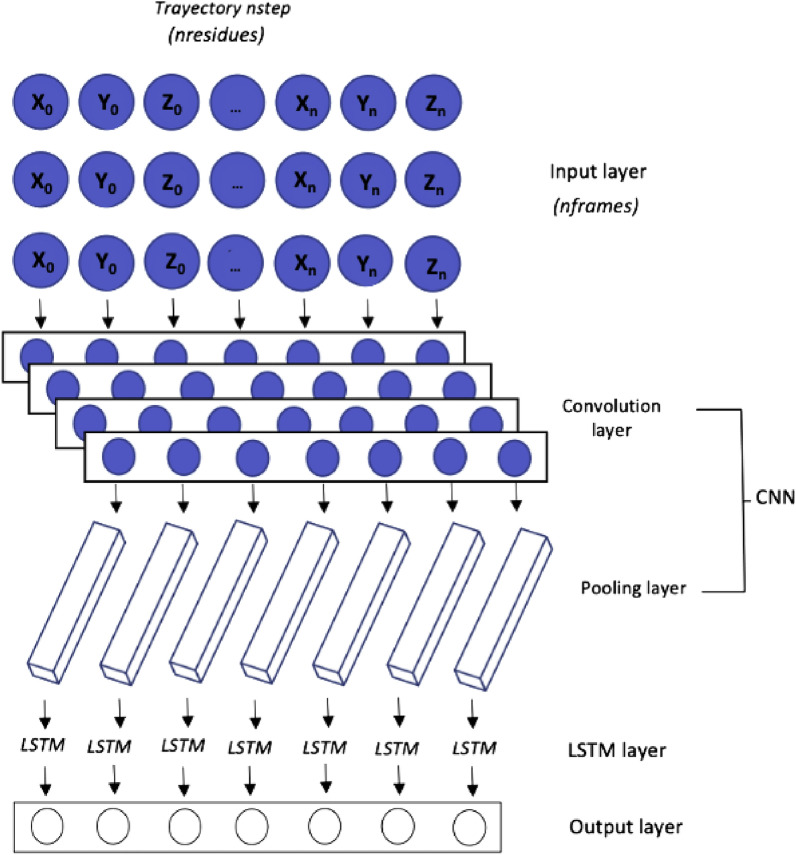
Illustration of CNN-LSTM chain of algorithms.* n-residues* represent the consecutive position of the amino acids in the protein.

The CNN is a feedforward Neural Network proposed by Lecun *et al.*^52^ that has been shown to perform exceedingly well in image and natural language processing tasks^53^. It can also be used effectively to predict time series. Local perception and weight sharing of the CNN model can dramatically reduce its number of parameters, increasing the effectiveness of its learning process^54^. The CNN architecture consists mostly of a convolution layer and a two-part regrouping layer. Each convolution layer contains a plurality of convolution kernels. Following the convolution operation of the convolution layer, the data features are extracted, but their dimension is very high, so, and to reduce the computational cost of training, a pooling layer is added after the convolution layer to reduce the feature dimension^55^. In our experiments, a combined CNN-LSTM shallow Neural Network-based forecasting model has also been applied. Figure 3 shows an schematic representation of such a model architecture, and Table 2 provides a brief quantitative summary of its elements.

**Table 2:** Deep neural networks architecture.

Layer (type)	Output shape	Parameter
ULSTM		
LSTM (LSTM)	(None, 100)	378800
Repeat vector (RepeatVector)	(None, 1, 100)	0
LSTM (LSTM)	(None, 1, 100)	80400
Time distributed (TimeDistributed)	(None, 1, 150)	85446
Total params		544,646
Trainable params		544,646
Non-trainable params		0
BLSTM		
Bidirectional (Bidirectional)	(None, 100)	417200
Repeat vector (RepeatVector)	(None, 1, 100)	0
Bidirectional (Bidirectional)	(None, 1, 100)	80400
Time distributed (TimeDistributed)	(None, 1, 150)	95142
Total params		592,742
Trainable params		592,742
Non-trainable params		0
CNN+LSTM		
Conv1D	(None, 5, 64)	487360
MaxPooling1D	(None, 3, 64)	0
Dropout	(None, 3, 64)	0
Conv1D	(None, 3, 128)	73856
MaxPooling 1D	(None, 2, 128)	0
Dropout	(None, 2, 128)	0
BatchNormalization	(None, 2, 128)	512
Flatten	(None, 256)	0
RepeatVector	(None, 1, 256)	0
lstm (LSTM)	(None, 1, 100)	142800
TimeDistributed	(None, 1, 846)	85446
Total params		789,974
Trainable params		789,718
Non-trainable params		256

### Experimental methodology

Data underwent linear max-min normalisation^56^ and were returned to the original range of values in Angstrom (Å) units. The 3D positions of amino acids were extracted for each frame. Note, though, that the original database included the positions of atoms, instead of the positions of amino acids. Therefore, the amino acids mass centers were calculated and used as 3D positions that represent them. Data prepossessing was performed on the 3D positions (x, y, z dimensions) of each residue. Five time frames were used to train the model, and the next frame was predicted from these. An overlap of 4 frames was used to select the following sequence on the training set; this means that, in the end, each frame was predicted, providing the 5 previous frames in the simulation. Then, the average error of all predictions was calculated and reported.

Two thousand trajectories per simulation were used. Simulations are represented in two states: ${\upbeta }$2AR-2RH1 (simulations started in inactive state) and ${\upbeta }$2AR-3P0G (simulations started in inactive state) both for *APO*, *Full Agonist* (BI-167107) and *partial inverse antagonist* conforming 6 types of the simulation. We refer to trajectories as *nClones*.

The LSTM model training was carried out using the 3D amino acid position (*x*, *y*, *z*) per frame. We refer to the length of a sequence as *nSteps-in* (which was 5 in our experiments), and the position of the amino acid representative data point as the *center of mass*. The centers of mass of the amino acid were employed as a representation of the residue’s position. This center of mass parameter is the best representative of the amino acid in the 3D space under LSTM forecasting^36^. All experiments were carried out in this way.

The parameters of training configuration of the LSTM were: epochs = 100, verbose = 0, activation = relu, input shape = (nSteps-in, length of amino acid chain). All the remaining parameters of the Keras^57^ framework were retained by default.

The RF^58^ algorithm, used for comparison, is a more conventional ML approach that can behave as a regression model. The Sklearn library^59^ implementation was used with default parameters, with the exception of maxdepth = number of residues 3 (*xyz* positions) and randomstate = 0.

For each type of simulation, 10,000 trajectories are available from the database reported in Kohlhoff et al.^38^. Two thousand of these are randomly selected and split into 5 folds (Four of them were used for cross-validation, and the remaining hold-out fold was used for test.) each including 400 trajectories. Results do not improve by using a bigger number of simulations. Ten iterations of this procedure were performed to obtain statistical significance tests.

The experiments were repeated by randomly choosing 2000 trajectories from the original set of trajectories (10,000). This procedure generates 10 models that have been evaluated and yields 10 RMSD values per experiment (ML approaches and the 6 types of simulations). Students t-tests (*pvalue* $< 0,001$ and *pvalue* $< 0,005$)^60^ were carried out to find statistically significant differences between experiments. Standard deviations and *p*-values are shown in the tables of results.

Given the potentially higher flexibility of the loops (ECL and ILC), a specific analysis was performed in which only the amino acids belonging to the TM were used for training and prediction. These results can then be compared with models obtained by training with the full protein sequences.

The quality of the test predictions was assessed through the Root Mean Square Deviation (RMSD), commonly used to assess the similarity between simulated and predicted atomic coordinates and therefore straightforwardly generalizable to the centers of mass of the amino acids, as we have have done in this study.

### Experimental setup

Three experiments were performed for both GPCR states. The first experiment (E1) evaluates the capability of the RF,ULSTM, BLSTM and CNN-LSTM models to predict steps of the GPCR trajectories, discriminating by TM, ECL and ICL regions for active and inactives states.

For that, the prediction error of each of the amino acid positions was calculated and compared between models, region by region. Similar evaluations were carried out in the second experiment (E2), this time focused on the seven TM, comparing the prediction error between the *2RH1* and *3P0G* for each TM (TM1 to TM6).

Focusing now on the large dynamics of the ICL and ECL of the GPCR, Experiment E3 evaluates the prediction capability of the models on the ICL (ICL1, ICL2) and ECL (ECL1, ECL2, ECL3) regions.

## Results and discussion

Our study investigates the capability of LSTM models to predict GPCR MD trajectories for its different states and constituent regions. Different GPCR regions may play different roles in the MD associated to each state. The investigation of regions individually is therefore relevant.

As mentioned, prediction errors are reported using the RMSD^61^, with original range values in Angstroms (Å) units. The standard deviation (*std*)^62^ between experiment repetitions is also calculated for the RMSD metric. Table 3 shows the RMSD and the *std* in Å for *2RH1* inactive and *3P0G* active states, in which prediction errors for RF, ULSTM, BLSTM, CNN-LSTM are compared. The results discriminated by APO, PA and PIA simulations are also shown in Table 3.

Regarding experiment E1, three questions can be answered:

- **Which model is the best one?** TM, ECL and ICL regions show similar prediction error values for the different models. Furthermore, these errors are not uniform when comparing APO, FA and PIA simulations (see italics in Table 3). Despite the increasing complexity of the models evaluated (RF, ULSTM, BLSTM and CNN-LSTM), no clear differences were found between them with the exception of RF, which performed significantly worse than the rest. Using an Occam’s razor criterion, ULSTM, being the simplest model in terms of architecture complexity and computational resources used to obtain this goals, should be selected.
- **Which GPCR region yields best results and in what analysis?** The TM regions are shown to be the best predicted regions of the GPCRs (see bold values in Table 3, with significant differences with $p<0.005$ for ${\upbeta }$2AR-2RH1 (inactive state in APO) and $p<0.001$ for the remaining simulations respect to ICL and ECL . Comparing now the ICL and ECL regions, ILC achieve the minimum error for APO, FA and PIA simulations, with the exception of the active state of the APO simulation, where the minimum error was obtained for the ECL region, however no significance different are shown between these regions.
- **Are there any substantial differences between active and inactive states?** Regarding inactive (2RH1) and active (3P0G) states, no strong differences were observed between their regions. Two exceptions are found in this analysis, once for the ECL region in the APO state and the TM region in the FA state that show significant differences (* pvalue *$< {0.05 }$), see RMSD values in Table 3 with single asterisk). Only in these cases, the active state show clear differences from the inactive one.

The experimental results show that the LSTM model performed the best in predicting the dynamics of the TM regions and that, overall, the ICL regions yielded the highest prediction error. However the dynamics of the GPCR are different by region determining that some regions are more prone to conformational changes. The flexibility of the molecule was calculated by region and compared by examining the prediction error between regions, as shown in Tables 3 and 6.

The transmembrane regions are less flexible than the ILC and EXL regions, which are more likely to experience changes in their 3D structure. This may be one of the reasons why minor error has been obtained in the transmembrane regions.

Regarding now E2 and focusing on the six TM regions, the prediction errors are shown in Tables 4 and 7. The former includes results obtained training the models with the whole receptor, while the latter includes results obtained training the models only with the TM regions. No statistically significant differences ($p>0.05$) are observed between the errors obtained for models trained with loops and those trained without loops. In general terms, TM2 was the best predicted region for the active state. These results coincide for all the simulations for APO, FA and PIA. However, for the inactive state, the best values were obtained for TM2 or TM3, depending on the simulation, showing substantial differences between both regions. TM2 and TM3 do not show statistically significant differences between them ($\textit{p}>0.05$). However, TM2 shows significant differences with respect to TM1, TM4, TM5, TM6, TM7 in almost all simulations and models (except in active state for PIA simulation with BLSTM in frot of TM4), see value ^∗1^ in Table 4.

A more detailed analysis of the experimental results provided further information on the MD of the specific receptor regions. While TM2 coincides for all the simulations as the best predicted in active and inactive states, TM3, instead, is the best for inactive states in PIA simulations, but it do not show significance (*pvalue* $> 0.05$)differences in contrast of TM2.

These proteins share a highly conserved motif of seven transmembrane helices connected by three extracellular and three intracellular loops. Movements of transmembrane regions III and IV are responsible for the activation of G protein-coupled receptors^63^. The conformational changes of the receptor transmembrane regions are closely related to the ${\upbeta }$2-adrenergic activation (${\upbeta }$2AR) pathway^64^. It is known that an outer displacement of TM6 from the centre of the helices and displacements of TM5 and TM7 are part of the activation mechanism of a receptor^10^. However, the details of the mechanisms of interaction between residues, which unchain the activation, are still unclear. The Helices 6 and 7 of the original simulation show a strong difference between inactive and active structures, with a relative displacement of 6.951 and 3.47 Å, respectively. Helices 1, 2, and 4 are shifted away from the center in the active state, while helix 3 is noticeably nearer (the range of relative displacements is from 1.4 to 2.277 Å). During simulation, helix 6 moves inwards in the active state simulations, while helix 7 moves outwards. When *Kohlhoff et al.,*^38^ compare simulations started from the active with those started from the inactive state, the relative displacements of helices 1 through 4 between active and inactive remain almost constant, indicating the importance of their rearrangement as a distinguishing element of receptor activation. Therefore, it is essential to carry out this experiment to examine each domain of the transmembrane region of the receptor in detail. It could easily be claimed that the region that is structurally incomplete is most inaccurately modelled.

The original paper describing the MD simulations used in this study did not attempt to model the missing sections, which becomes a limitation of our reported results. Regarding missing sections in ICL (231-262 in 2RH1, and 228-264 in 3P0G between helices 5 and 6), it is difficult to draw solid conclusions about the differences between ICL and ECL since the protein is not accurately modelled. Nevertheless, ${\upbeta }$2AR remains functional even in the absence of ICL3.

The prediction errors for experiment E3 are shown in Tables 5 for ICL and 6 for ECL. For the ICL regions, ICL1 was identified as the region with the lowest prediction error in both states and all simulations. Interestingly , the accurate prediction of MD of the ICL1 region contrasts with the results of ICL2, which yields a significantly statistics differences (*pvalue *$< 0.01$), with RMSD differences greater than 0.2 Å.

In the case of the ECL regions, both simulations showed that ECL1 had the lowest prediction error, which was significantly different from ICL2 (*pvalue* $< 0.05$) and from ECL3 (*pvalue* $< 0.01$). These results coincide for APO and FA simulations (see the RMSD values in Table 6). Regarding the PIA simulations, only significant differences (*pvalue* $< 0.05$) were found between ECL1 and ECL2 (Table 7).

Beyond that, our experiments were carried out with three ML models of increasing structural complexity in terms of their network architecture. The results show inconclusive differences between DL models, with minor differences depending on the simulations and state. Therefore, the simplest of these models, namely ULSTM, would be the preferred choice for further investigation. In the comparison of DL models (ULSTM, BLSTM, CNN-LSTM) with conventional ML models (RF), DL models have shown strong and significant (*pvalue* $< 0.01$) differences with RF.

The existing literature has reported the use of the RMSD error in the *static* 3D prediction for different proteins: for instance, Chen & Brooks^65^ used it as a metric to ascertain whether MD simulations provide high-resolution refinement of protein structure. Lee *et al.*^66^ predicted 3D structure using molecular mechanics based on the surface area free energies for two small proteins (HP-36 and S15). The RMSD error values obtained were 0.77 Å for HP-36 protein and 0.83 Å for S15 protein. More specifically for GPCRs, Kaczor *et al.*^67^ analysed different methods for protein-protein docking and evaluated the generation of new digital protein-protein complexes in the transmembrane environment. The best method achieved an overall RMSD lower than 0.7 Å in 8 out of 12 simulations. Even if not directly comparable to this study, we have reported errors lower than 0.13 Å as measured by RMSD in simulations of dynamics that are a major challenge for models.

**Figure 4:**
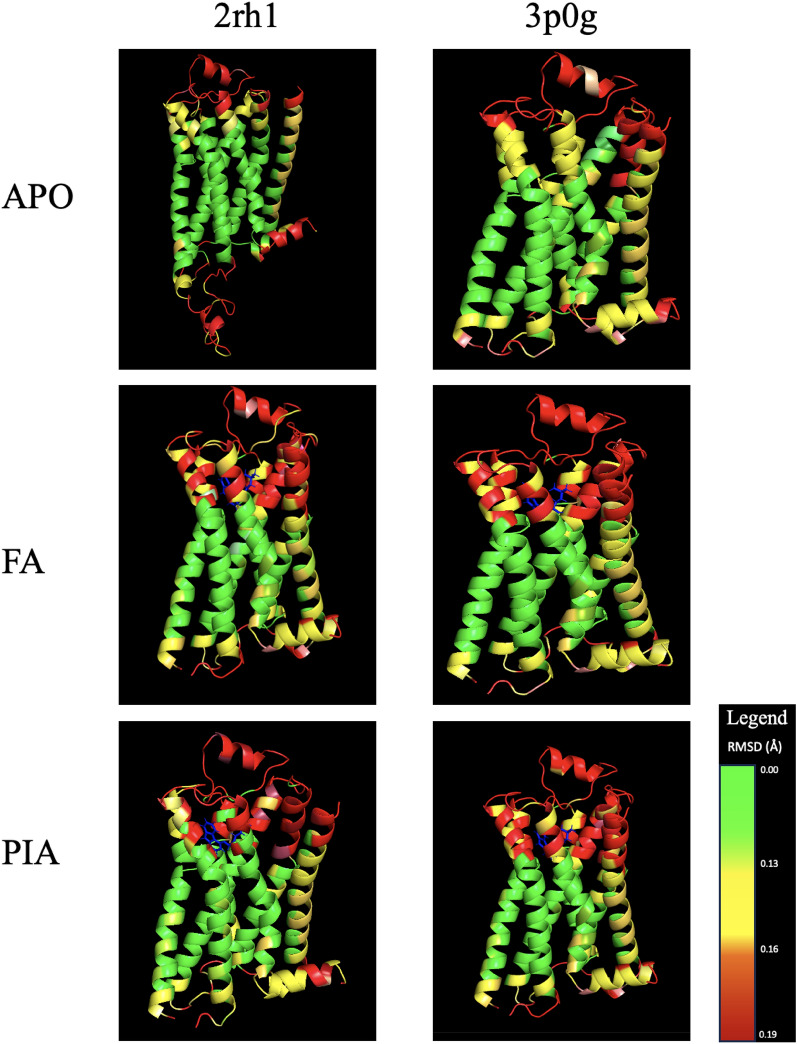
Colour-coded mapping of the average prediction errors (as measured by RMSD in Å) of LSTM models for the ${\upbeta }$2 adrenergic GPCR for TM, ECL and ICL regions, in columns, and for APO, Full Agonist (FA) and Partial Inverse Antagonist (PIA) in rows. Blue colour represent the ligand bound to the protein.

The approach proposed in this paper allows predicting 3D residue positions from the MD time series. It could be used in prospective experiments by setting threshold error targets for the discrimination between states and exploring whether the method can achieve them and how well they compare with those obtained with alternative methods. Furthermore, such investigation could be assisted by visualising results by residue, as in Figure 4, which maps prediction errors using coloured ribbons. This would allow for visual interactive and intuitive evaluation, assessing which are the best or worst modelled residues, distinguishing those that are exposed to the solvent, or those exposed to the ligand, to name a few possibilities.

**Table 3:** Prediction error of the RF, ULSTM, BLSTM and CNN-LSTM models, as measured by RMSD and *std* in Å units for TM, ICL, ECL loops of the APO, FA, PIA simulations for *2rh1* and *3p0g* states.

Region	MD-RMSD	RF	ULSTM	BLSTM	CNN-LSTM
APO	2rh1				
TM ^∗+^	2.0120	0.2660 ^∗∗-^ ${\pm }$ 0.0040	**0.1390** ${\pm }$ 0.0080	0.1460 ${\pm }$ 0.0080	0.1430 ${\pm }$ 0.0020
ICL	2.3200	0.3150 ^∗∗-^ ${\pm }$ 0.0090	0.1790 ${\pm }$ 0.0090	0.1910 ${\pm }$ 0.0070	0.1830 ${\pm }$ 0.0019
ECL	2.0800	0.2770 ^∗∗-^ ${\pm }$ 0.0020	0.1810 ${\pm }$ 0.0090	0.1850 ${\pm }$ 0.0080	0.1880 ${\pm }$ 0.0020
APO	3p0g				
TM ^∗∗+^	1.8652	0.1746 ^∗∗-^ ${\pm }$ 0.0019	0.1438 ${\pm }$ 0.0052	0.1444 ${\pm }$ 0.0041	**0.1387** ${\pm }$ 0.0012
ICL	1.5981	0.2213 ^∗∗-^ ${\pm }$ 0.0027	0.1887 ${\pm }$ 0.0056	0.1920 ${\pm }$ 0.0056	0.1878 ${\pm }$ 0.0019
ECL	1.5306	0.2101 ^∗∗-^ ${\pm }$ 0.0010	0.1781 ${\pm }$ 0.0054	0.1791 ${\pm }$ 0.0042	0.1725 ${\pm }$ 0.0011
FA	2rh1				
TM ^∗∗+^	1.1757	0.1672 ^∗-^ ${\pm }$ 0.0021	0.1350 ${\pm }$ 0.0042	0.1358 ${\pm }$ 0.0041	**0.1349** ${\pm }$ 0.0042
ICL	1.1518	0.2533 ^∗∗-^ ${\pm }$ 0.0033	0.1823 ${\pm }$ 0.0041	0.1850 ${\pm }$ 0.0041	0.1856 ${\pm }$ 0.0043
ECL	1.1476	0.2157 ^∗∗-^ ${\pm }$ 0.0038	0.1713 ${\pm }$ 0.0056	0.1705 ${\pm }$ 0.0054	0.1716 ${\pm }$ 0.0050
FA	3p0g				
TM ^∗∗+^	1.7808	0.1736 ^∗∗-^ ${\pm }$ 0.0021	0.1362 ${\pm }$ 0.0052	0.1343 ${\pm }$ 0.0036	**0.1230** ${\pm }$ 0.0023
ICL	1.4829	0.2132 ^∗∗-^ ${\pm }$ 0.0022	0.1884 ${\pm }$ 0.0059	0.1863 ${\pm }$ 0.0045	0.1834 ${\pm }$ 0.0031
ECL	1.4983	0.2071 ^∗∗-^ ${\pm }$ 0.0024	0.1744 ${\pm }$ 0.0066	0.1737 ${\pm }$ 0.0047	0.1682 ${\pm }$ 0.0029
PIA	2rh1				
TM ^∗∗+^	1.7686	0.1782 ^∗∗-^ ${\pm }$ 0.0043	0.1388 ${\pm }$ 0.0029	0.1388 ${\pm }$ 0.0028	**0.1338** ${\pm }$ 0.0066
ICL	1.5180	0.2712 ^∗∗-^ ${\pm }$ 0.0073	0.1896 ${\pm }$ 0.0026	0.1889 ${\pm }$ 0.0046	0.1850 ${\pm }$ 0.0092
ECL	1.4525	0.2134 ^∗∗-^ ${\pm }$ 0.0027	0.1725 ${\pm }$ 0.0035	0.1715 ${\pm }$ 0.0032	0.1675 ${\pm }$ 0.0061
PIA	3p0g				
TM ^∗∗+^	1.7853	0.1888 ^∗∗-^ ${\pm }$ 0.0047	0.1365 ${\pm }$ 0.0071	**0.1298** ${\pm }$ 0.0053	0.1324 ${\pm }$ 0.0016
ICL	1.4847	0.2260 ^∗∗-^ ${\pm }$ 0.0048	0.1921 ${\pm }$ 0.0075	0.1856 ${\pm }$ 0.0065	0.1890 ${\pm }$ 0.0018
ECL	1.4513	0.2188 ^∗∗-^ ${\pm }$ 0.0029	0.1675 ${\pm }$ 0.0082	0.1591 ${\pm }$ 0.0063	0.1623 ${\pm }$ 0.0020
Mean					
TM ^∗∗+^	1.7313	0.1914 ^∗∗-^ ${\pm }$ 0.0032	0.1382 ${\pm }$ 0.0054	0.1382 ${\pm }$ 0.0046	**0.1343** ${\pm }$ 0.0003
ICL	1.5926	0.0025 ^∗∗-^ ${\pm }$ 0.0049	0.1867 ${\pm }$ 0.0058	0.1881 ${\pm }$ 0.0054	0.1856 ${\pm }$ 0.0037
ECL	1.5267	0.2237 ^∗∗-^ ${\pm }$ 0.0025	0.1741 ${\pm }$ 0.0064	0.1731 ${\pm }$ 0.0053	0.1717 ${\pm }$ 0.0032

Statistics of significantly worse^(-)^ and significantly better^(+)^ differences with *p* $$< 0.05^{*}$$ and *p*$$< 0.01^{**}$$ are included. The symbols in the first column indicate significantly better differences between regions, while in the model results columns indicate significantly worse differences between models. The *Mean* row at the bottom of the table indicates mean errors across regions. Bold values show the minimum error accross regions and models.

**Table 4:** Prediction error of the RF, ULSTM, BLSTM and CNN-LSTM models, as measured by RMSD metric in Å units. The six transmembrane regions (TM) regions of the APO, FA and PIA simulations for *2rh1* and *3p0g* states.

Region	MD-RMSD	RF	USLTM	BLSTM	CNN-LSTM
APO	2rh1				
TM1	2.6361	0.2328^∗∗-^ ${\pm }$ 0.0056	0.1480 ${\pm }$ 0.0080	0.1563 ${\pm }$ 0.0068	0.1518 ${\pm }$ 0.0024
TM2^∗+^	1.5520	0.1998^∗∗-^ ${\pm }$ 0.0025	**0.1313** ${\pm }$ 0.0075	0.1387 ${\pm }$ 0.0058	0.1350 ${\pm }$ 0.0025
TM3	1.5807	0.2143^∗∗-^ ${\pm }$ 0.0026	0.1325 ${\pm }$ 0.0077	0.1389 ${\pm }$ 0.0059	0.1364 ${\pm }$ 0.0020
TM4	1.7552	0.2090^∗∗-^ ${\pm }$ 0.0023	0.1416 ${\pm }$ 0.0068	0.1482 ${\pm }$ 0.0056	0.1472 ${\pm }$ 0.0026
TM5	1.9092	0.2300^∗∗-^ ${\pm }$ 0.0055	0.1455 ${\pm }$ 0.0084	0.1510 ${\pm }$ 0.0067	0.1501 ${\pm }$ 0.0020
TM6	1.7479	0.5320^∗∗-^ ${\pm }$ 0.0032	0.1372 ${\pm }$ 0.0153	0.1437 ${\pm }$ 0.0192	0.1412 ${\pm }$ 0.0052
TM7	1.7785	0.2451^∗∗-^ ${\pm }$ 0.0071	0.1396 ${\pm }$ 0.0072	0.1485 ${\pm }$ 0.0063	0.1428 ${\pm }$ 0.0017
APO	3p0g				
TM1	2.1303	0.1921^∗∗-^ ${\pm }$ 0.0009	0.1480 ${\pm }$ 0.0053	0.1490 ${\pm }$ 0.0039	0.1427 ${\pm }$ 0.0011
TM2^∗+^	1.6303	0.1694^∗∗-^ ${\pm }$ 0.0032	0.1340 ${\pm }$ 0.0048	0.1349 ${\pm }$ 0.0040	**0.1270** ${\pm }$ 0.0015
TM3	1.5573	0.1585^∗∗-^ ${\pm }$ 0.0022	0.1370 ${\pm }$ 0.0048	0.1378 ${\pm }$ 0.0035	0.1321 ${\pm }$ 0.0012
TM4	1.6851	0.1874^∗∗-^ ${\pm }$ 0.0043	0.1433 ${\pm }$ 0.0046	0.1420 ${\pm }$ 0.0045	0.1377 ${\pm }$ 0.0026
TM5	1.8959	0.1693^∗∗-^ ${\pm }$ 0.0015	0.1514 ${\pm }$ 0.0058	0.1520 ${\pm }$ 0.0040	0.1475 ${\pm }$ 0.0013
TM6	1.7132	0.1730^∗∗-^ ${\pm }$ 0.0011	0.1517 ${\pm }$ 0.0056	0.1527 ${\pm }$ 0.0042	0.1469 ${\pm }$ 0.0011
TM7	1.6891	0.1726^∗∗-^ ${\pm }$ 0.0004	0.1680 ${\pm }$ 0.0053	0.1632 ${\pm }$ 0.0040	0.1516 ${\pm }$ 0.0009
FA	2rh1				
TM1	2.2130	0.1632^∗∗-^ ${\pm }$ 0.0014	0.1472 ${\pm }$ 0.0042	0.1488 ${\pm }$ 0.0042	0.1478 ${\pm }$ 0.0045
TM2^∗+^	1.1630	0.1485^∗∗-^ ${\pm }$ 0.0018	0.1276 ${\pm }$ 0.0040	0.1279 ${\pm }$ 0.0037	0.1269 ${\pm }$ 0.0044
TM3	1.1557	0.1651^∗∗-^ ${\pm }$ 0.0034	0.1296 ${\pm }$ 0.0040	**0.1261** ${\pm }$ 0.0039	0.1262 ${\pm }$ 0.0044
TM4	1.1685	0.1794^∗∗-^ ${\pm }$ 0.0027	0.1325 ${\pm }$ 0.0046	0.1331 ${\pm }$ 0.0042	0.1326 ${\pm }$ 0.0041
TM5	1.1896	0.1866^∗∗-^ ${\pm }$ 0.0013	0.1435 ${\pm }$ 0.0039	0.1449 ${\pm }$ 0.0041	0.1436 ${\pm }$ 0.0044
TM6	1.1713	0.1604^∗∗-^ ${\pm }$ 0.0021	0.1370 ${\pm }$ 0.0034	0.1380 ${\pm }$ 0.0032	0.1366 ${\pm }$ 0.0042
TM7	1.1689	0.1773^∗∗-^ ${\pm }$ 0.0031	0.1476 ${\pm }$ 0.0040	0.1442 ${\pm }$ 0.0036	0.1396 ${\pm }$ 0.0040
FA	3p0g				
TM1	2.4546	0.1934^∗∗-^ ${\pm }$ 0.0020	0.1418 ${\pm }$ 0.0054	0.1346 ${\pm }$ 0.0166	0.1355 ${\pm }$ 0.0027
TM2^∗+^	1.5985	0.1680^∗∗-^ ${\pm }$ 0.0035	0.1269 ${\pm }$ 0.0046	0.1255 ${\pm }$ 0.0033	**0.1205** ${\pm }$ 0.0029
TM3	1.5074	0.1653^∗∗-^ ${\pm }$ 0.0035	0.1314 ${\pm }$ 0.0047	0.1295 ${\pm }$ 0.0030	0.1248 ${\pm }$ 0.0027
TM4	1.7063	0.1796^∗∗-^ ${\pm }$ 0.0015	0.1367 ${\pm }$ 0.0047	0.1345 ${\pm }$ 0.0034	0.1312 ${\pm }$ 0.0024
TM5	1.8776	0.1660^∗∗-^ ${\pm }$ 0.0011	0.1405 ${\pm }$ 0.0052	0.1376 ${\pm }$ 0.0037	0.1337 ${\pm }$ 0.0020
TM6	1.6032	0.1616^∗∗-^ ${\pm }$ 0.0019	0.1450 ${\pm }$ 0.0053	0.1432 ${\pm }$ 0.0041	0.1390 ${\pm }$ 0.0021
TM7	1.7178	0.1816^∗∗-^ ${\pm }$ 0.0015	0.1412 ${\pm }$ 0.0052	0.1420 ${\pm }$ 0.0038	0.1357 ${\pm }$ 0.0022
PIA	2rh1				
TM1	2.1959	0.1714^∗∗-^ ${\pm }$ 0.0048	0.1493 ${\pm }$ 0.0216	0.1493 ${\pm }$ 0.0222	0.1434 ${\pm }$ 0.0219
TM2^∗+^	1.6432	0.1768^∗∗-^ ${\pm }$ 0.0057	0.1295 ${\pm }$ 0.0156	0.1299 ${\pm }$ 0.0162	0.1248 ${\pm }$ 0.0159
TM3	1.5364	0.1687^∗∗-^ ${\pm }$ 0.0017	0.1293 ${\pm }$ 0.0214	0.1294 ${\pm }$ 0.0226	**0.1246** ${\pm }$ 0.0219
TM4	1.6747	0.1789^∗∗-^ ${\pm }$ 0.0053	0.1355 ${\pm }$ 0.0136	0.1365 ${\pm }$ 0.0151	0.1326 ${\pm }$ 0.0144
TM5	1.7934	0.1918^∗∗-^ ${\pm }$ 0.0029	0.1482 ${\pm }$ 0.0342	0.1477 ${\pm }$ 0.0358	0.1427 ${\pm }$ 0.0333
TM6	1.7762	0.1819^∗∗-^ ${\pm }$ 0.0055	0.1436 ${\pm }$ 0.0259	0.1428 ${\pm }$ 0.0265	0.1380 ${\pm }$ 0.0259
TM7	1.7943	0.1936^∗∗-^ ${\pm }$ 0.0087	0.1595 ${\pm }$ 0.0106	0.1505 ${\pm }$ 0.0089	0.14314 ${\pm }$ 0.0124
PIA	3p0g				
TM1	2.6085	0.1978^∗∗-^ ${\pm }$ 0.0047	0.1466 ${\pm }$ 0.0074	0.1401 ${\pm }$ 0.0054	0.1417 ${\pm }$ 0.0121
TM2^∗+^	1.6066	0.1819^∗∗-^ ${\pm }$ 0.0027	0.1291 ${\pm }$ 0.0071	**0.1220** ${\pm }$ 0.0049	0.1244 ${\pm }$ 0.0016
TM3	1.5384	0.1656^∗∗-^ ${\pm }$ 0.0042	0.1297 ${\pm }$ 0.0069	0.1221^∗1^ ${\pm }$ 0.0048	0.1253 ${\pm }$ 0.0019
TM4	1.6484	0.2159^∗∗-^ ${\pm }$ 0.0098	0.1338 ${\pm }$ 0.0068	0.1259 ${\pm }$ 0.0049	0.1291 ${\pm }$ 0.0020
TM5	1.8164	0.1845^∗∗-^ ${\pm }$ 0.0039	0.0068 ${\pm }$ 0.0266	0.1315 ${\pm }$ 0.0053	0.1353 ${\pm }$ 0.0026
TM6	1.5573	0.1838^∗∗-^ ${\pm }$ 0.0034	0.1457 ${\pm }$ 0.0064	0.1393 ${\pm }$ 0.0050	0.1421 ${\pm }$ 0.0020
TM7	1.7212	0.1919^∗∗-^ ${\pm }$ 0.0040	0.1482 ${\pm }$ 0.0064	0.1424 ${\pm }$ 0.0050	0.1464 ${\pm }$ 0.0020
Mean					
TM1	2.3731	0.1918^∗∗-^ ${\pm }$ 0.0032	0.1468 ${\pm }$ 0.0086	0.1247 ${\pm }$ 0.0098	0.1438 ${\pm }$ 0.0075
TM2^∗+^	1.5323	0.1741^∗∗-^ ${\pm }$ 0.0032	0.1297 ${\pm }$ 0.0073	0.1298 ${\pm }$ 0.0063	**0.1264** ${\pm }$ 0.0048
TM3	1.4793	0.1729^∗∗-^ ${\pm }$ 0.0029	0.1316 ${\pm }$ 0.0083	0.1316 ${\pm }$ 0.0073	0.1292 ${\pm }$ 0.0057
TM4	1.6064	0.1917^∗∗-^ ${\pm }$ 0.0043	0.1372 ${\pm }$ 0.0068	0.1367 ${\pm }$ 0.0063	0.1351 ${\pm }$ 0.0047
TM5	1.7470	0.1880^∗∗-^ ${\pm }$ 0.0027	0.1326 ${\pm }$ 0.0140	0.1441 ${\pm }$ 0.0099	0.1421 ${\pm }$ 0.0076
TM6	1.5949	0.2321^∗∗-^ ${\pm }$ 0.0029	0.1434 ${\pm }$ 0.0103	0.1433 ${\pm }$ 0.0104	0.1406 ${\pm }$ 0.0067
TM7	1.6151	0.1937^∗∗-^ ${\pm }$ 0.0032	0.1489 ${\pm }$ 0.0056	0.1481 ${\pm }$ 0.0045	0.1432 ${\pm }$ 0.0022

Statistics of significantly worse^(-)^ and significantly better^(+)^ differences with *p* $$< 0.05^{*}$$ and *p*$$< 0.01^{**}$$ are included. The symbols in the first column indicate significantly better differences between TM regions, while, in the model results columns, they indicate significantly worse differences between models. The *Mean* row at the bottom indicates mean errors across regions. Bold values show the minimum error accross regions and models. Symbol ^∗1^ refers to no significant differences as compared to TM2.

**Table 5:** Prediction error of the RF, ULSTM, BLSTM and CNN-LSTM models, as measured by RMSD in Å units add std on the tables. The intracellular loops (ICL) of the APO, FA, PIA simulations for *2rh1* and *3p0g* states.

Region	MD-RMSD	RF	ULSTM	BLSTM	CNN-LSTM
APO	2rh1				
ICL1^∗∗+^	1.6690	0.2769^∗∗-^ ${\pm }$ 0.0122	**0.1669** ${\pm }$ 0.0085	0.1727 ${\pm }$ 0.0068	0.1699${\pm }$ 0.0026
ICL2	1.9260	0.3104^∗∗-^ ${\pm }$ 0.0135	0.1926 ${\pm }$ 0.0091	0.1980 ${\pm }$ 0.0069	0.1978 ${\pm }$ 0.0025
APO	3p0g				
ICL1^∗∗+^	1.7470	0.1869^∗-^ ${\pm }$ 0.0038	0.1747 ${\pm }$ 0.0057	0.1777 ${\pm }$ 0.0055	**0.1717** ${\pm }$ 0.0017
ICL2	2.0260	0.2183^∗-^ ${\pm }$ 0.0009	0.2026 ${\pm }$ 0.0057	0.2062 ${\pm }$ 0.0057	0.2038 ${\pm }$ 0.0022
FA	2rh1				
ICL1^∗∗+^	1.7960	0.1945^∗∗-^ ${\pm }$ 0.0024	**0.1713** ${\pm }$ 0.0039	0.1725 ${\pm }$ 0.0038	0.1732 ${\pm }$ 0.0046
ICL2	1.3420	0.2466^∗∗-^ ${\pm }$ 0.0033	0.1934 ${\pm }$ 0.0042	0.1978 ${\pm }$ 0.0043	0.1979 ${\pm }$ 0.0042
FA	3p0g				
ICL1^∗∗+^	1.7510	0.1828^∗-^ ${\pm }$ 0.0031	0.1751 ${\pm }$ 0.0050	0.1739 ${\pm }$ 0.0038	**0.1704** ${\pm }$ 0.0034
ICL2	2.0170	0.2269^∗∗-^ ${\pm }$ 0.0019	0.2017 ${\pm }$ 0.0065	0.1988 ${\pm }$ 0.0050	0.1964 ${\pm }$ 0.0032
PIA	2rh1				
ICL1^∗∗+^	1.7770	0.2270^∗∗-^ ${\pm }$ 0.0085	0.1777 ${\pm }$ 0.0022	0.1766 ${\pm }$ 0.0045	**0.1703** ${\pm }$ 0.0102
ICL2	2.0020	0.2590^∗∗-^ ${\pm }$ 0.0080	0.2002 ${\pm }$ 0.0030	0.2027 ${\pm }$ 0.0047	0.1996 ${\pm }$ 0.0087
PIA	3p0g				
ICL1^∗∗+^	1.7460	0.1896^∗-^ ${\pm }$ 0.0053	**0.1746** ${\pm }$ 0.0078	0.1795 ${\pm }$ 0.0062	0.1766 ${\pm }$ 0.0020
ICL2	1.9670	0.2520^∗∗-^ ${\pm }$ 0.0040	0.1967 ${\pm }$ 0.0074	0.2049 ${\pm }$ 0.0068	0.2013 ${\pm }$ 0.0023
Mean					
ICL1^∗∗+^	1.7477	0.2096^∗∗-^ ${\pm }$ 0.0059	0.1747 ${\pm }$ 0.0049	0.1755 ${\pm }$ 0.0051	**0.1720** ${\pm }$ 0.0041
ICL2	1.8800	0.2522^∗∗-^ ${\pm }$ 0.0053	0.1989 ${\pm }$ 0.0054	0.2014 ${\pm }$ 0.0056	0.1995 ${\pm }$ 0.0038

Statistics of significantly worse^(-)^ and significantly better^(+)^ differences with *p* $$< 0.05^{*}$$ and *p*$$< 0.01^{**}$$. The symbols in the first column indicate significantly better differences between ICL regions, while, in the model results columns, they indicate significantly worse differences between models. The *Mean* row at the bottom of the table indicates mean errors across regions. Bold values show the minimum error accross regions and models.

**Table 6:** Prediction error of the RF, ULSTM, BLSTM and CNN-LSTM models, as measured by RMSD metric in Å units. The three extracellular loops (ECL) of the APO, FA, PIA simulations for *2rh1* and *3p0g* states.

Region	MD-RMSD	RF	ULSTM	BLSTM	CNN-LSTM
APO	2rh1				
ECL1^∗∗+^	2.0800	0.2440^∗∗-^ ${\pm }$ 0.0078	**0.1750** ${\pm }$ 0.0084	0.1850 ${\pm }$ 0.0078	0.1808 ${\pm }$ 0.0029
ECL2	2.2200	0.2230^∗∗-^ ${\pm }$ 0.0064	0.1840 ${\pm }$ 0.0091	0.1930 ${\pm }$ 0.0084	0.1920 ${\pm }$ 0.0016
ECL3	2.4500	0.2400^∗∗-^ ${\pm }$ 0.0033	0.1930 ${\pm }$ 0.0089	0.1990 ${\pm }$ 0.0064	0.1971 ${\pm }$ 0.0022
APO	3p0g				
ECL1^∗∗+^	1.4983	0.1935^∗∗-^ ${\pm }$ 0.0005	0.1702 ${\pm }$ 0.0049	0.1718 ${\pm }$ 0.0021	**0.1641** ${\pm }$ 0.0014
ECL2	1.5356	0.1996^∗-^ ${\pm }$ 0.0056	0.1821 ${\pm }$ 0.0055	0.1828 ${\pm }$ 0.0047	0.1767 ${\pm }$ 0.0012
ECL3	1.3340	0.2003^∗∗-^ ${\pm }$ 0.0071	0.1929 ${\pm }$ 0.0060	0.1949 ${\pm }$ 0.0046	0.1895${\pm }$ 0.0015
FA	2rh1				
ECL1^∗+^	1.4525	0.1962^∗∗-^ ${\pm }$ 0.0028	0.1681 ${\pm }$ 0.0060	**0.1679** ${\pm }$ 0.0054	0.1692 ${\pm }$ 0.0054
ECL2	1.8524	0.2064^∗∗-^ ${\pm }$ 0.0065	0.1730 ${\pm }$ 0.0059	0.1720 ${\pm }$ 0.0058	0.1728 ${\pm }$ 0.0053
ECL3	1.4563	0.2133^∗∗-^ ${\pm }$ 0.0033	0.1823 ${\pm }$ 0.0041	0.1819 ${\pm }$ 0.0041	0.1816 ${\pm }$ 0.0040
FA	3p0g				
ECL1^∗∗+^	1.4513	0.2119^∗∗-^ ${\pm }$ 0.0041	0.1679 ${\pm }$ 0.0061	0.1682 ${\pm }$ 0.0033	**0.1618** ${\pm }$ 0.0031
ECL2	1.6783	0.2340^∗∗-^ ${\pm }$ 0.0012	0.1776 ${\pm }$ 0.0070	0.1765 ${\pm }$ 0.0051	0.1715 ${\pm }$ 0.0031
ECL3	1.7785	0.2157^∗∗-^ ${\pm }$ 0.0022	0.1860 ${\pm }$ 0.0066	0.1845 ${\pm }$ 0.0059	0.1806 ${\pm }$ 0.0023
PIA	2rh1				
ECL1^∗+^	1.9613	0.1934^∗∗-^ ${\pm }$ 0.0026	0.1711 ${\pm }$ 0.0039	0.1690 ${\pm }$ 0.0027	**0.1664** ${\pm }$ 0.0066
ECL2	2.3271	0.2017^∗∗-^ ${\pm }$ 0.0019	0.1732 ${\pm }$ 0.0033	0.1727 ${\pm }$ 0.0033	0.1680 ${\pm }$ 0.0063
ECL3	2.5942	0.2235^∗∗-^ ${\pm }$ 0.0022	0.1918 ${\pm }$ 0.0040	0.1893 ${\pm }$ 0.0035	0.1831 ${\pm }$ 0.0055
PIA	3p0g				
ECL1^∗+^	2.1627	0.1945^∗∗-^ ${\pm }$ 0.0021	0.1647 ${\pm }$ 0.1793	**0.1577** ${\pm }$ 0.1771	0.1604 ${\pm }$ 0.1563
ECL2	2.0666	0.2137^∗∗-^ ${\pm }$ 0.0022	0.1690 ${\pm }$ 0.2008	0.1598 ${\pm }$ 0.1974	0.1633 ${\pm }$ 0.1773
ECL3	2.8734	0.2234^∗∗-^ ${\pm }$ 0.0025	0.1924 ${\pm }$ 0.2146	0.1843 ${\pm }$ 0.2212	0.1878 ${\pm }$ 0.2042
Mean					
ECL1^∗+^	1.7677	0.2056^∗∗-^ ${\pm }$ 0.0033	0.1695 ${\pm }$ 0.0348	0.1699 ${\pm }$ 0.0331	**0.1671** ${\pm }$ 0.0293
ECL2	1.9467	0.2131^∗∗-^ ${\pm }$ 0.0040	0.1765 ${\pm }$ 0.0386	0.1761 ${\pm }$ 0.0374	0.1741 ${\pm }$ 0.0325
ECL3	2.0811	0.2194^∗∗-^ ${\pm }$ 0.0034	0.1897 ${\pm }$ 0.0407	0.1890 ${\pm }$ 0.0410	0.1866 ${\pm }$ 0.0366

Statistics of significantly worse^(-)^ and significantly better^(+)^ differences with *p* $$< 0.05^{*}$$ and *p*$$< 0.01^{**}$$ have been included. The symbols in the first column indicate significantly better differences between ECL regions, while, in the model results columns, they indicate significantly worse differences between models. The *Mean* row at the bottom indicates mean errors across regions. Bold values show the minimum accross regions and models.

**Table 7:** *Predictionerrorofthe* RF, ULSTM, BLSTM and CNN-LSTM models trained only with TM regions (and, therefore, to be compared with results in Table 4), as measured by RMSD metric in Å units. The six transmembrane regions (TM) regions of the APO, FA and PIA simulations for *2rh1* and *3p0g* states.

Region	MD-RMSD	RF	ULSTM	BLSTM	CNN-LSTM
APO	2rh1				
TM1	2.6361	0.2374^∗∗-^ ${\pm }$ 0.0061	0.1506 ${\pm }$ 0.0086	0.1549 ${\pm }$ 0.0071	0.1499 ${\pm }$ 0.0025
TM2^∗∗+^	1.5390	0.2001^∗∗-^ ${\pm }$ 0.0036	**0.1304** ${\pm }$ 0.0083	0.1406 ${\pm }$ 0.0070	0.1401 ${\pm }$ 0.0027
TM3	1.5807	0.2208^∗∗-^ ${\pm }$ 0.0026	0.1385 ${\pm }$ 0.0063	0.1343 ${\pm }$ 0.0059	0.1360 ${\pm }$ 0.0032
TM4	1.7552	0.2106^∗∗-^ ${\pm }$ 0.0038	0.1448 ${\pm }$ 0.0020	0.1505 ${\pm }$ 0.0057	0.1560 ${\pm }$ 0.0012
TM5	1.9092	0.2420^∗∗-^ ${\pm }$ 0.0035	0.1399 ${\pm }$ 0.0033	0.1489 ${\pm }$ 0.0052	0.1509 ${\pm }$ 0.0030
TM6	1.7479	0.1882^∗∗-^ ${\pm }$ 0.0034	0.1402 ${\pm }$ 0.0140	0.1423 ${\pm }$ 0.0182	0.1413 ${\pm }$ 0.0049
TM7	1.7785	0.2942^∗∗-^ ${\pm }$ 0.0065	0.1424 ${\pm }$ 0.0068	0.1472 ${\pm }$ 0.0051	0.1422 ${\pm }$ 0.0009
APO	3p0g				
TM1	2.1303	0.1864^∗∗-^ ${\pm }$ 0.0004	0.1491 ${\pm }$ 0.0073	0.1458 ${\pm }$ 0.0023	0.1412 ${\pm }$ 0.0013
TM2^∗∗+^	1.6303	0.1591^∗∗-^ ${\pm }$ 0.0038	0.1343 ${\pm }$ 0.0014	0.1399 ${\pm }$ 0.0071	**0.1298** ${\pm }$ 0.0017
TM3	1.5573	0.1604^∗-^ ${\pm }$ 0.0082	0.1393 ${\pm }$ 0.0052	0.1343 ${\pm }$ 0.0010	0.1365 ${\pm }$ 0.0024
TM4	1.6851	0.1838^∗∗-^ ${\pm }$ 0.0088	0.1474 ${\pm }$ 0.0012	0.1406 ${\pm }$ 0.0003	0.1396 ${\pm }$ 0.0066
TM5	1.8959	0.1636^∗-^ ${\pm }$ 0.0013	0.1585 ${\pm }$ 0.0022	0.1521 ${\pm }$ 0.0084	0.1510 ${\pm }$ 0.0078
TM6	1.7132	0.1745^∗∗-^ ${\pm }$ 0.0016	0.1504 ${\pm }$ 0.0031	0.1451 ${\pm }$ 0.0062	0.1410 ${\pm }$ 0.0067
TM7	1.6891	0.1710^∗-^ ${\pm }$ 0.0089	0.1696 ${\pm }$ 0.0090	0.1600 ${\pm }$ 0.0011	0.1553 ${\pm }$ 0.0079
FA	2rh1				
TM1	2.2130	0.1721^∗∗-^ ${\pm }$ 0.0019	0.1342 ${\pm }$ 0.0069	0.1389 ${\pm }$ 0.0035	0.1466 ${\pm }$ 0.0033
TM2^∗+^	1.1630	0.1310^∗-^ ${\pm }$ 0.0020	**0.1200** ${\pm }$ 0.0033	0.1301 ${\pm }$ 0.0029	0.1264 ${\pm }$ 0.0014
TM3	1.1557	0.1734^∗∗-^ ${\pm }$ 0.0010	0.1246 ${\pm }$ 0.0043	0.1244 ${\pm }$ 0.0096	0.1310 ${\pm }$ 0.0069
TM4	1.1685	0.1812^∗∗-^ ${\pm }$ 0.0024	0.1295 ${\pm }$ 0.0081	0.1299 ${\pm }$ 0.0042	0.1390 ${\pm }$ 0.0067
TM5	1.1896	0.1856^∗∗-^ ${\pm }$ 0.0030	0.1389 ${\pm }$ 0.0034	0.1377 ${\pm }$ 0.0076	0.1344 ${\pm }$ 0.0086
TM6	1.1713	0.1598^∗∗-^ ${\pm }$ 0.0036	0.1344 ${\pm }$ 0.0014	0.1402 ${\pm }$ 0.0032	0.1400 ${\pm }$ 0.0037
TM7	1.1689	0.1789^∗∗-^ ${\pm }$ 0.0039	0.1506 ${\pm }$ 0.0050	0.1388 ${\pm }$ 0.0056	0.1422 ${\pm }$ 0.0031
FA	3p0g				
TM1	2.4546	0.1907^∗∗-^ ${\pm }$ 0.0019	0.1432 ${\pm }$ 0.0058	0.1334 ${\pm }$ 0.0028	0.1348 ${\pm }$ 0.0063
TM2^∗+^	1.5985	0.1539^∗∗-^ ${\pm }$ 0.0033	0.1246 ${\pm }$ 0.0056	0.1276 ${\pm }$ 0.0030	**0.1191** ${\pm }$ 0.0012
TM3	1.5074	0.1711^∗∗-^ ${\pm }$ 0.0043	0.1276 ${\pm }$ 0.0074	0.1302 ${\pm }$ 0.0038	0.1272 ${\pm }$ 0.0099
TM4	1.7063	0.1744^∗∗-^ ${\pm }$ 0.0076	0.1396 ${\pm }$ 0.0042	0.1388 ${\pm }$ 0.0065	0.1329 ${\pm }$ 0.0096
TM5	1.8776	0.1720^∗∗-^ ${\pm }$ 0.0012	0.1377 ${\pm }$ 0.0093	0.1377 ${\pm }$ 0.0020	0.1312 ${\pm }$ 0.0008
TM6	1.6032	0.1674^∗∗-^ ${\pm }$ 0.0011	0.1463 ${\pm }$ 0.0080	0.1479 ${\pm }$ 0.0038	0.1379 ${\pm }$ 0.0027
TM7	1.7178	0.1822 ${\pm }$ 0.0049	0.1517 ${\pm }$ 0.0034	0.1370 ${\pm }$ 0.0011	0.1347 ${\pm }$ 0.0010
PIA	2rh1				
TM1	2.1959	0.1801^∗∗-^ ${\pm }$ 0.0054	0.1392 ${\pm }$ 0.0081	0.1505 ${\pm }$ 0.0020	0.1464 ${\pm }$ 0.0104
TM2^∗+^	1.6432	0.1704^∗∗-^ ${\pm }$ 0.0034	0.1241 ${\pm }$ 0.0088	0.1276 ${\pm }$ 0.0017	**0.1220** ${\pm }$ 0.0100
TM3	1.5364	0.1693^∗∗-^ ${\pm }$ 0.0021	0.1287 ${\pm }$ 0.0079	0.1299 ${\pm }$ 0.0108	0.1262 ${\pm }$ 0.0087
TM4	1.6747	0.1792^∗∗-^ ${\pm }$ 0.0062	0.1488 ${\pm }$ 0.0093	0.1310 ${\pm }$ 0.0066	0.1317 ${\pm }$ 0.0098
TM5	1.7934	0.1867^∗∗-^ ${\pm }$ 0.0066	0.1450 ${\pm }$ 0.0090	0.1478 ${\pm }$ 0.0093	0.1469 ${\pm }$ 0.0053
TM6	1.7762	0.1828^∗∗-^ ${\pm }$ 0.0070	0.1472 ${\pm }$ 0.0101	0.1488 ${\pm }$ 0.0082	0.1399 ${\pm }$ 0.0024
TM7	1.7976	0.1918^∗∗-^ ${\pm }$ 0.0075	0.1603 ${\pm }$ 0.0096	0.1711 ${\pm }$ 0.0076	0.15111 ${\pm }$ 0.0223
PIA	3p0g				
TM1	2.6085	0.1887^∗∗-^ ${\pm }$ 0.0035	0.1403 ${\pm }$ 0.0035	0.1557 ${\pm }$ 0.0072	0.1443 ${\pm }$ 0.0101
TM2^∗+^	1.6066	0.1822^∗∗-^ ${\pm }$ 0.0090	0.1286 ${\pm }$ 0.0044	**0.1237** ${\pm }$ 0.0039	0.1257 ${\pm }$ 0.0016
TM3	1.5384	0.1697^∗∗-^ ${\pm }$ 0.0032	0.1308 ${\pm }$ 0.0066	0.1295 ${\pm }$ 0.0077	0.1301 ${\pm }$ 0.0095
TM4	1.6484	0.2089^∗∗-^ ${\pm }$ 0.0020	0.1360 ${\pm }$ 0.0036	0.1310 ${\pm }$ 0.0059	0.1268 ${\pm }$ 0.0071
TM5	1.8164	0.1897^∗∗-^ ${\pm }$ 0.0025	0.1424 ${\pm }$ 0.0072	0.1398 ${\pm }$ 0.0040	0.1382 ${\pm }$ 0.0065
TM6	1.5573	0.1824^∗∗-^ ${\pm }$ 0.0090	0.1407 ${\pm }$ 0.0054	0.1334 ${\pm }$ 0.0093	0.1442 ${\pm }$ 0.0028
TM7	1.7212	0.1892^∗∗-^ ${\pm }$ 0.0007	0.1433 ${\pm }$ 0.0073	0.1486 ${\pm }$ 0.0027	0.1432 ${\pm }$ 0.0047
Mean					
TM1	2.3730	0.1925^∗∗-^ ${\pm }$ 0.0032	0.1427 ${\pm }$ 0.0067	0.1465 ${\pm }$0.0041	0.1438 ${\pm }$ 0.0056
TM2^∗+^	1.5301	0.1661^∗∗-^ ${\pm }$ 0.0041	**0.1270** ${\pm }$ 0.0053	0.1315 ${\pm }$0.004	0.1271 ${\pm }$ 0.0031
TM3	1.4793	0.1774^∗∗-^ ${\pm }$ 0.0035	0.1315 ${\pm }$ 0.0062	0.1304 ${\pm }$0.0064	0.1311 ${\pm }$ 0.0067
TM4	1.6063	0.1896^∗∗-^ ${\pm }$ 0.0051	0.1410 ${\pm }$ 0.0047	0.1369 ${\pm }$0.0048	0.1376 ${\pm }$ 0.0068
TM5	1.7470	0.1899^∗∗-^ ${\pm }$ 0.0030	0.1437 ${\pm }$ 0.0057	0.1440 ${\pm }$0.0060	0.1421 ${\pm }$ 0.0053
TM6	1.5948	0.2591^∗∗-^ ${\pm }$ 0.0042	0.1432 ${\pm }$ 0.0070	0.1429 ${\pm }$0.0081	0.1407 ${\pm }$ 0.0038
TM7	1.6455	0.2012^∗∗-^ ${\pm }$ 0.0054	0.1529 ${\pm }$ 0.0068	0.1504 ${\pm }$0.0038	0.1447 ${\pm }$ 0.0066

Statistics of significantly worse^(-)^ and significantly better^(+)^ differences with *p* $$< 0.05^{*}$$ and *p*$$< 0.01^{**}$$ have been included. The symbols in the first column indicate significantly better differences between TM regions, while, in the model results columns, they indicate significantly worse differences between models. The *Mean* row at the bottom indicates mean errors across regions. Bold values show the minimum error between regions and models.

## Conclusions

LSTM Neural Networks have in the past shown promise in problems of GPCR dynamics forecasting. The current study has provided evidence that LSTM models, in three different architectures, are capable of predicting the dynamic trajectories of GPCRs in six states with a reasonable efficacy, and far better that more standard ML models such as RF.

The TM helices are a key GPCR region due to their physiological role in signal transmission. Our LSTM models have been abble to predict the dynamics of TM2 and TM3 the best. Nevertheless, the details of the mechanism of interaction between amino-acids that unchains the activation remain unclear.

Although ULSTM is the shallowest of the investigated DL architectures, it has yielded competitive performance when compared to more complex models such as BLSTM and the combination CNN-LSTM.

LSTM models, though, suffer from some limitations when used to process long MD trajectories. For this reason, as a next step, we plan to investigate the capabilities of generative models (which have successfully been used for the modelling of protein MDs^16^) such as Transformers^68^ or Autoencoders^69^, for the prediction of long trajectories. We also plan to evaluate alternative representations of GPCR data, including graph representations.

No significant differences were observed between models trained with loops and only with the TM regions. This could be due to the fact that the data representation used by the models is a frame-by-frame relationship. We suggest that further research should be conducted on the representation of molecular dynamics through graphs that explicitly consider the connections between neighbouring residues.

## Data Availability

The data that support the findings of this study are available from Kohlhoff *et al.*^38^, but restrictions apply to their availability, which were used under license for the current study. Data are however available from the authors upon reasonable request^38^.
