# Author Correction: GPCR molecular dynamics forecasting using recurrent neural networks

**DOI:** 10.1038/s41598-024-60566-w

**Published:** 2024-04-30

**Authors:** Juan Manuel López-Correa, Caroline König, Alfredo Vellido

**Affiliations:** 1 Universitat Politècnica de Catalunya, Barcelona, Spain; 2 IDEAI-UPC - Research Center, Universitat Politècnica de Catalunya, Barcelona, Spain

Correction to: *Scientific Reports* 10.1038/s41598-023-48346-4, published online 28 November 2023

The original version of this Article contained an error in the Acknowledgements section.

“This work is funded by Spanish PID2019-104551RB-I00 research project and by the PRE2020-092428 Ph.D. training program, through the Ministry of Science and Innovation.”

now reads

“This work is funded by the Spanish PID2022-143299OB-I00 research project and by the PRE2020-092428 PhD training program, through the Ministry of Science and Innovation.”

The original Article has been corrected.

